# The influence of vision on sound localization abilities in both the horizontal and vertical planes

**DOI:** 10.3389/fpsyg.2013.00932

**Published:** 2013-12-12

**Authors:** Vanessa Tabry, Robert J. Zatorre, Patrice Voss

**Affiliations:** ^1^Department of Neurology and Neurosurgery, Montreal Neurological Institute, McGill UniversityMontreal, QC, Canada; ^2^International Laboratory for Brain, Music and Sound Research (BRAMS)Montreal, QC, Canada

**Keywords:** sound localization, vision, pointing methods, spatial hearing, blindness

## Abstract

Numerous recent reports have suggested that individuals deprived of vision are able to develop heightened auditory spatial abilities. However, most such studies have compared the blind to blindfolded sighted individuals, a procedure that might introduce a strong performance bias. Indeed, while blind individuals have had their whole lives to adapt to this condition, sighted individuals might be put at a severe disadvantage when having to localize sounds without visual input. To address this unknown, we compared the sound localization ability of eight sighted individuals with and without a blindfold in a hemi-anechoic chamber. Sound stimuli were broadband noise delivered via two speaker arrays: a horizontal array with 25 loudspeakers (ranging from −90° to +90°; 7.5°) and a vertical array with 16 loudspeakers (ranging from −45° to +67.5°). A factorial design was used, where we compared two vision conditions (blindfold vs. non-blindfold), two sound planes (horizontal vs. vertical) and two pointing methods (hand vs. head). Results show that all three factors significantly interact with one another with regards to the average absolute deviation error. Although blindfolding significantly affected all conditions, it did more so for head-pointing in the horizontal plane. Moreover, blindfolding was found to increase the tendency to undershoot more eccentric spatial positions for head-pointing, but not hand-pointing. Overall, these findings suggest that while proprioceptive cues appear to be sufficient for accurate hand pointing in the absence of visual feedback, head pointing relies more heavily on visual cues in order to provide a precise response. It also strongly argues against the use of head pointing methodologies with blindfolded sighted individuals, particularly in the horizontal plane, as it likely introduces a bias when comparing them to blind individuals.

## Introduction

It has been proposed that the blind compensate for their lack of vision by sharpening their auditory abilities (Niemeyer and Starlinger, 1981; Muchnick et al., 1991; Gougoux et al., 2004). In particular, auditory spatial processing has been a topic of particular interest due to its high relevance for spatial navigation. There have been multiple reports of enhanced sound localization abilities in early blind humans (Ashmead et al., 1998; Lessard et al., 1998; Doucet et al., 2005; Gougoux et al., 2005) as well as enhanced auditory spatial discrimination abilities (Röder et al., 1999; Voss et al., 2004) in the horizontal (azimuthal) plane. Other findings, however, point to degraded auditory spatial abilities when having to localize sounds in the vertical plane (Zwiers et al., 2001; Lewald, 2002). Aside from the obvious difference in auditory spatial planes studied, another important potential source for this discrepancy relates to the use of different pointing methods. While the studies reporting enhancements typically used hand pointing procedures to measure subjects, the latter used either head pointing (Zwiers et al., 2001) or a swivel pointer that was fixed in front of the subjects (Lewald, 2002). Overall, these findings raise interesting questions on how the visual status of an individual interacts with other factors such as the auditory spatial plane and the pointing method used when having to localize sounds in the environment.

The selection of an appropriate pointing method in sound localization studies comparing the sighted to the blind should therefore be given careful attention, because the two subpopulations may differ in their proficiency in using the same pointing method (e.g., hand pointing or head pointing). This is an issue of particular importance because in most studies comparing the sighted and the blind, the sighted are transiently visually deprived, which may hamper their ability to use a pointing method to localize a target. On the other hand, the early blind may be more proficient with the pointing method, having developed non-visual compensatory mechanisms to orient body parts toward specific directions. As such, potential differences in pointing ability may partially account for previously shown differences in sound localization performance between the two groups. Further, vision is more heavily weighted comparatively to proprioception in judgments requiring multisensory integration, and so exerts a strong bias on proprioception (Hay et al., 1965; Pick and Warren, 1969; Rossetti et al., 1995). Indeed, while sighted children show a decrease in the relative importance of proprioception in multisensory integration with age, blind children do not, likely because in their case, vision does not become the dominant localizing modality, as it does in the sighted (Pick and Warren, 1969). Additionally, the directive control of vision over proprioception has been shown to increase with long-term visual experience (Birch and Lefford, 1963). Consequently, both populations may differentially rely on proprioceptive cues when having to explicitly localize sound sources; not to mention that the reliance on such cues could differ depending on the pointing method (head vs. hand).

To better ascertain the relative sound localization abilities of the sighted and blind, it is vital to identify pointing methods whose accuracy are as little affected as possible by transient or developmental visual deprivation, in order to isolate and reduce potential biases in the responses that are unrelated to spatial sound perception. In the current study, we addressed the issue of whether transient visual deprivation of sighted individuals (i.e., removal of visual feedback cues) would differentially affect different pointing methods. We also assessed whether the lack of visual feedback would have a differential effect on localization in orthogonal sound planes (vertical vs. horizontal). To address these questions we used a 2 × 2 × 2 factorial design, where we compared two visual conditions (blindfold vs. non-blindfold), two pointing methods (hand pointing vs. head pointing) and two auditory spatial planes (horizontal vs. vertical). We predicted main effects of visual condition where performance would be best without the blindfold, and of auditory spatial plane given the higher auditory spatial resolution of the human auditory system in the horizontal plane (see Makous and Middlebrooks, 1990). While we did not necessarily expect a main effect of pointing method (see Haber et al., 1993), we were particularly interested in determining if possible interaction effects could exist between the visual condition and pointing method given the different proprioceptive cues that underlie head and hand pointing. Similarly, we predicted an interaction between visual condition and auditory spatial plane, where blindfolding would have a greater effect on performance in the vertical plane given the poorer performance of blind individuals in the vertical plane (Zwiers et al., 2001; Lewald, 2002).

## Materials and methods

### Participants

The participants were eight right-handed sighted volunteers (four male, mean age: 22 ± 2.98 years), with no history of neurological disease. They gave their written informed consent in accordance with guidelines approved by the Montreal Neurological Institute (MNI) and the *Centre de Recherche Interdisciplinaire en Réadaptation* (CRIR), and received monetary compensation for participating. Each participant was tested in two separate 1-h long sessions that were approximately 1 week apart. The participants have self-reported normal or corrected-to-normal vision. Standard audiometric assessments were performed for all participants and indicated normal and comparable hearing in both ears.

### Conditions

Three variables were manipulated for each subject when having to localize sounds: visual condition (blindfold vs. no blindfold), pointing method (head pointing vs. hand pointing), and auditory spatial plane (horizontal vs. vertical). As a result of this 2 × 2 × 2 factorial design, each subject performed the task under eight conditions, which were counterbalanced across all subjects. Trial runs were completed over two separate testing sessions that were held approximately 1 week apart.

### Materials and stimuli

Sound localization tests were controlled by a custom-designed Matlab script (r.2009a; MathWorks) and stimuli were generated using TDT System 3 (Tucker-Davis-Technology). The stimuli consisted of 100 ms pink noise bursts (10 ms rise/fall times) presented at 60 dB SPL as measured at the center of the array.

**Figure 1:**
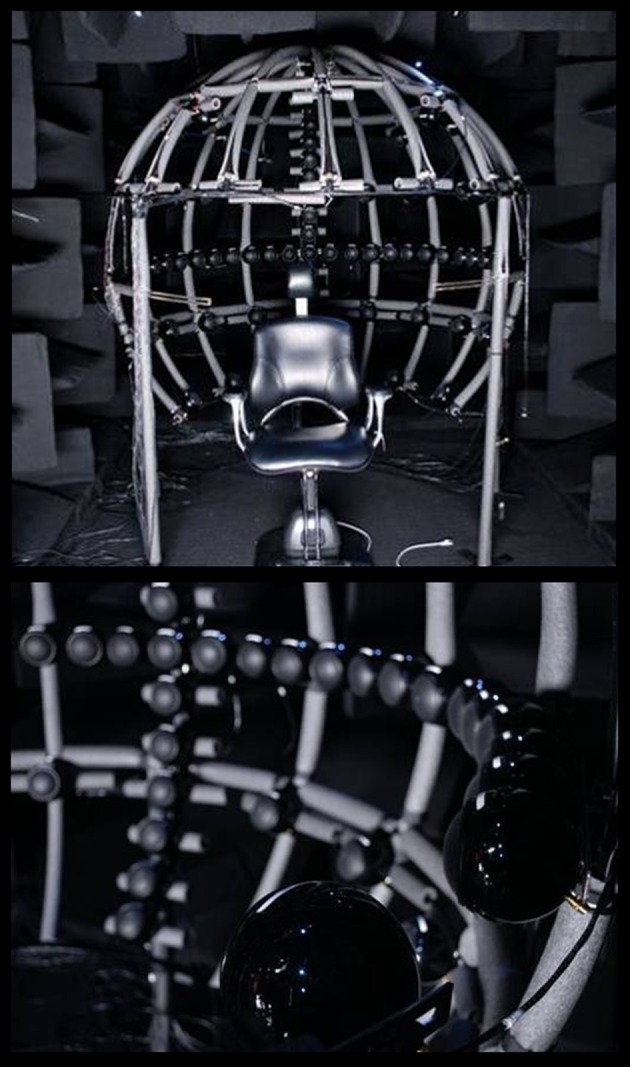
**Sound localization setup**. Illustrated here is the hemi-anechoic chamber and the acoustic apparatus used to test sound localization. The bottom panel provides a close-up of the arrays of loudspeakers along the horizontal and vertical midlines. The additional speakers were not used in the current experiment.

The experiment was carried out in a hemi-anechoic chamber (2.5 × 5.5 × 2.5 m). The acoustic apparatus used to test sound localization consisted of 25 loudspeakers on the horizontal plane and 16 on the vertical plane, mounted on two semicircular railings with a radius of 90 cm (see Figure 1). Each location was sampled four times in each of the eight experimental conditions. The positions of the loudspeakers ranged from −90 to +90° on the horizontal plane, and from −37.5 to +67.5° on the vertical plane; thus providing a spatial resolution of 7.5° on both planes. Subjects were seated such that the speakers in the horizontal plane were positioned at ear level and those in the vertical plane were aligned with the subjects' mid-sagittal plane. The loudspeaker located at the crossing of both railings was therefore located at 0° azimuth, 0° elevation. The loudspeakers were hidden by a thin black cotton sleeve in such a way that the distance to the speakers could be seen, but not their spacing, size, or exact location. In addition, two fabric rulers were put in place along the semicircular railings; this was done so that an experimenter present could note laser-pointed locations (see procedure).

### Procedure

Subjects were seated in a fixed chair in front of the two semi-circular railings and were required to indicate the location of short noise bursts delivered through a randomly selected loudspeaker. Subjects were also instructed to maintain a head position pointing straight ahead until the end of the stimulus presentation, and were required to return to that position prior to starting the next trial (failure to do so would result in the inability to start the next trial; see also “*Recording method*” below for more details). Prior to beginning the experimental conditions, subjects performed practice trials until they felt at ease with the recording apparatus (typically 10–15 trials). They were also given short breaks when needed between trial blocks. Subjects were allowed to turn their shoulders if necessary when indicating peripheral sources. No headrest was mounted on the chair, in order to reduce the probability of obstructing head movements to extreme spatial locations. Trials were run in blocks of either horizontal or vertical trials. In each block the error was only computed in one dimension (either horizontal or vertical) in accordance with the auditory plane being tested, and the subjects always knew in advance which plane was being tested prior to starting each block.

### Recording method

#### Head-tracking apparatus

Subjects wore an elastic cap with a magnetic receiver of a 3D digitizer system (ISOTRAK II, Polhemus) that recorded the head position, and that was mounted with a laser pointer directing its beam straight ahead. Prior to each trial subjects were instructed to face the crossing point of both axes (0° azimuth, 0° elevation) and to record their head position with a button-press on a remote once they were satisfied with the position of the head. Following a trial, subjects were required to return to their initial position (centered on 0° azimuth, 0° elevation) and press the button on the remote. When the head was properly positioned, a brief high-frequency tone was played via the speaker directly above the head to indicate a correct head position, and was followed by the sound burst to be localized. In the event of an improper head positioning, a lower-frequency tone would be played and the subject was required to reposition their head appropriately.

#### Head pointing

As mentioned above, a laser pointer was mounted onto the subjects' heads along with the magnetic receiver of the digitizer system. When localizing a sound burst, subjects were instructed to orient their heads so that their noses pointed toward the perceived location of the sound source, and to hold still for a moment until an experimenter in the room could note the pointed location.

#### Hand pointing

Following the sound bursts, subjects were asked to point to its location with a hand held laser-pointer (in their dominant hand; all right-handed). The location was again marked down by an experimenter present in the room.

### Analysis

Three different dependant variables were entered into separate 2 (visual condition: blindfold vs. no blindfold) × 2 (pointing method: head pointing vs. hand pointing) × 2 (auditory spatial plane: horizontal vs. vertical) repeated measures ANOVAs: average overall unsigned error, average signed error and slope of the regression curve of the signed error as a function of the target location in space. The *unsigned error* consisted of the average absolute deviation (in degrees) of the response from the target location, irrespective of whether responses were undershooting or overshooting the target, and was taken to be a measure of overall accuracy. The *signed error* consisted in the average signed deviation from target, and was taken to indicate potential directional response biases (e.g., tendency to present a leftward or rightward shift in the horizontal plane). Lastly, the *slope* of the regression curve served as indicator of how the signed error varied as a function of target eccentricity.

## Results

Single trials with absolute errors that were larger than 3 standard deviations above the mean deviation per target location were considered outliers and removed from our analysis. As such, 0.75% of the total number of trials (*n* = 10496) were excluded. An additional 0.27% of the trials were discarded due to the subjects not holding the laser in position long enough for the experimenter to take note of the position.

### Absolute error

The main effect of visual condition was found to be significant, as subjects localized sounds more accurately without the blindfold [*F*_(1, 7)_ = 25.84, *p* < 0.001]. The main effect of auditory spatial plane was also significant, as horizontal sources were located more accurately then vertical ones [*F*_(1, 7)_ = 32.15, *p* < 0.001]. The main effect of pointing method was however non-significant [*F*_(1, 7)_ = 0.60, *p* = 0.465]. The *auditory plane* × *pointing method* interaction was also found to be significant [*F*_(1, 7)_ = 17.69, *p* = 0.004]. We then broke down the interaction into components by looking at the simple effects of each condition. This revealed that performance on the horizontal plane was better for hand-pointing than for head-pointing (*p* = 0.026), whereas head-pointing was better than hand-pointing on the vertical plane (*p* = 0.035).

**Figure 2:**
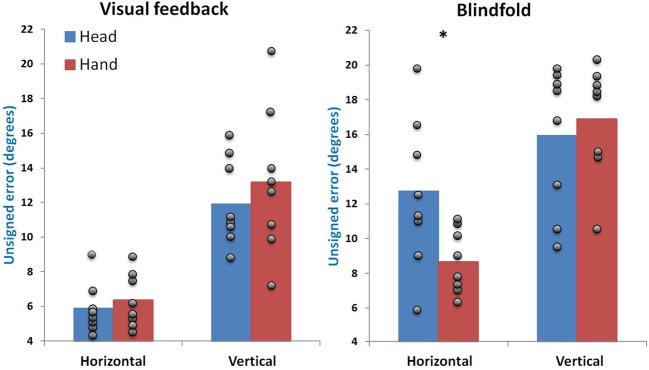
**Triple interaction**. Shown here is the significant interaction effect on the unsigned error between all three independent variables. Error bars represent the standard error of the mean. The gray dots represent the average localization error for each subject under each condition and illustrate the strong variability between subjects, particularly for the blindfolded conditions. The asterisk (^*^) indicates a significant difference between pointing methods for a given auditory plane and visual condition (*p* < 0.05).

Both the *auditory plane* × *visual condition* [*F*_(1, 7)_ = 0.26, *p* = 0.625] and the *pointing method* × *visual condition* [*F*_(1, 7)_ = 1.80, *p* = 0.222] interactions were found to be non-significant. However, a significant triple interaction was found between the effects of the pointing method, the visual condition and the auditory spatial plane on sound localization performance (*F*_(1, 7)_ = 6.45; *p* = 0.039). When examining the simple effects (illustrated in Figure 2), it was found that this interaction is primarily driven by the fact that the pointing methods do not differ from one another in most conditions (all *p* > 0.3), with the exception of the *blindfold-horizontal* conditions where head pointing was significantly less accurate than hand pointing (*p* = 0.029). The effect of blindfolding was however significant for all conditions [*hand-horizontal* (*p* = 0.019), *head-horizontal* (*p* = 0.009), *hand-vertical* (*p* = 0.047), *head-vertical* (*p* = 0.025)]. The effect was nonetheless greater for head pointing in the horizontal plane, where the average absolute error increased by 6.9°; all other blindfold-related increases were of 4.0° or less (see Figure 2).

### Signed error

**Figure 3:**
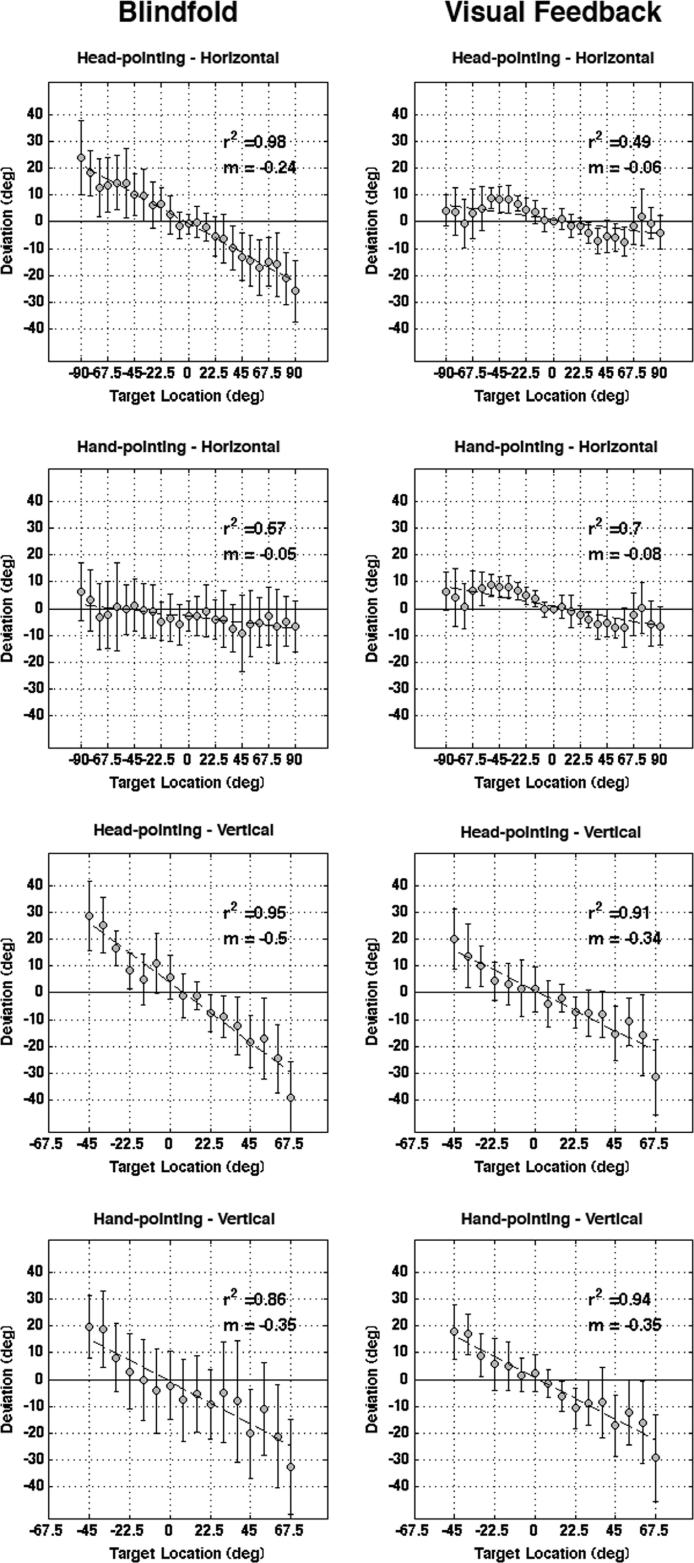
**Signed error plots**. Illustrated here are the signed error plots for each condition as a function of target location. Overlaid on top of the plots are first-order regression curves that were fitted to the signed error plots, for which the slopes can be taken as an index of the tendency to undershoot or overshoot target locations. In general, signed error tended to increase (undershoot) as a function of target eccentricity, and was further increased by blindfolding. However, this effect was primarily driven by the head pointing trials, as blindfolding did not have a significant effect on hand pointing. There was also a significant effect of auditory spatial plane, where the slope was greater for trials on the vertical plane.

Figure 3 shows the mean signed error in all conditions. A repeated measures 2 × 2 × 2 was performed on the signed error in the same manner as it was for the unsigned error. No main effects or interactions were found to be significant (all *p* > 0.146).

### Regression slope

As can be seen in Figure 3, signed error tended to increase as a function of target eccentricity and subjects tended to undershoot target locations. To address potential differences across the conditions, first-order regression curves were fitted to the signed error plots as a function of target location (see also Figure 3). These slopes can be taken as an index of the tendency to undershoot or overshoot target locations. We performed a similar 2 × 2 × 2 ANOVA to those above, but this time using the regression slope as the dependant measure. There was a significant main effect of visual condition, where the slope was steeper for blindfolded trials [*F*_(1, 7)_ = 6.87, *p* = 0.034], and of auditory spatial plane [*F*_(1, 7)_ = 40.25, *p* < 0.001], where the slope was steeper for the vertical plane. There was also a main effect of pointing [*F*_(1, 7)_ = 6.27, *p* = 0.041], where the slope for head pointing was found to be steeper than for hand pointing. However, a *visual condition* × *pointing method* interaction was also found to be significant [*F*_(1, 7)_ = 18.59, *p* = 0.004]. This effect was due to the fact that while blindfolding had no significant effect on the slope when hand-pointing (*p* = 0.341), it had a significant effect on it when head-pointing (*p* = 0.005). Accordingly, the slope associated with each pointing method did not differ with visual feedback (*p* = 0.215), whereas it was steeper for head pointing when blindfolded (*p* = 0.006). Overall, these results indicate that blindfolding increases the tendency to undershoot target locations for head-pointing only, and not hand-pointing. All other interaction effects failed to reach significance (all *p* > 0.314).

## Discussion

The purpose of the present study was primarily to investigate the effects of blindfolding and choice of pointing method on the sound localization performance of sighted individuals. In addition to the oft-studied horizontal plane, we included sound localization tasks presented along the vertical median plane, which generally requires individuals to use a different set of localization cues (Batteau, 1967; Gardner and Gardner, 1973). The addition of the vertical plane was done to ascertain whether blindfolding or the choice of pointing method would have a differential effect on the two auditory planes. Results showed that all three factors significantly interact with one another with regards to the average absolute localization error. Although blindfolding significantly affected all conditions, it did more so for head-pointing in the horizontal plane. Moreover, blindfolding was found to increase the tendency to undershoot more eccentric spatial positions for head-pointing, but not hand-pointing.

### Effect of auditory plane and pointing method

As expected, sound locations on the horizontal plane were more accurately localized than those on the vertical plane, where there is a strong tendency to undershoot the source locations (see Figure 3). This is highly consistent with previous findings demonstrating that auditory spatial resolution is far greater in the horizontal plane than in the vertical one (Oldfield and Parker, 1984; Makous and Middlebrooks, 1990). Although there was no global difference between the pointing methods, they provided different levels of accuracy with respect to the planes in which sounds were presented, as evidenced by the *auditory plane* × *pointing method* interaction. It was found that head-pointing was more accurate for localizing vertical targets (by approximately 1°), whereas hand pointing was more accurate for localization in the horizontal plane (by approximately 2°). However, a triple interaction revealed that this effect was primarily driven by the blindfolded conditions (discussed further below). In the conditions where visual feedback was available, the two pointing methods were not significantly different from one another (see Figure 2). While there is little comparative data for localization in the vertical plane, this result is consistent with previous findings indicating that both methods are comparable to one another for localization in the horizontal plane (Haber et al., 1993; Majdak et al., 2010). Although, there was a significant effect of pointing method on the slope of the regression curve, this was also driven by the effect of blindfolding, as the slope did not differ between pointing methods when visual feedback was available.

### Effect of blindfolding

The presence of visual feedback was found to lead to a significantly lower absolute localization error compared to performance on the same task when blindfolded. Although blindfolding increased this error for both pointing methods and for both auditory planes, this effect was greater for head pointing conditions, and was especially strong for head pointing in the horizontal plane (see Figure 2). Blindfolding also significantly increased the amount of undershooting for head-pointing (particularly for eccentric spatial positions), but not for hand pointing (as reflected by the regression curves slope seen in Figure 3). Overall, these effects of visual feedback on head pointing in the horizontal plane are highly consistent with the findings of Lewald et al. (2000), who showed that localizing with the head in darkness reduced localization accuracy and increased the tendency to undershoot target locations in the horizontal plane.

Pointing to sound sources in normal visual conditions arguably requires the combined and weighted processing of visual and proprioceptive cues. Indeed, matching a target position with the hand is better performed while having access to both visual and proprioceptive cues than with either modality alone (van Beers et al., 1999). Here we showed that blindfolding significantly increased the absolute localization error for both auditory spatial planes and both pointing methods. However, in the horizontal plane, the effect of blindfolding was shown to be greater for head than for hand pointing. Overall, our results suggest a greater dependence on visual cues for orienting one's head toward a specific location in space than for orienting one's arm. Indeed, blindfolding was shown to significantly affect both the average deviation from the target (in the horizontal plane) and the tendency to undershoot them more so for head-pointing trials.

So why would blindfolding (i.e., the removal of visual input and feedback) affect both pointing methods differently in the horizontal plane but not in the vertical plane? One possible explanation stems from the fact that the most peripheral positions in the vertical condition weren't as eccentric as those used in the horizontal plane; however this is also true for hand pointing conditions and therefore seems like an unlikely cause of the discrepancy. An alternative point of view could be that both pointing methods should be considered more or less equal (as evidenced in three of the four conditions), and that blindfolding for some reason induces a more pronounced effect specifically on head pointing in the horizontal plane. Why this is the case is also unclear. One possibility is the existence of different underlying physical restrictions in rotating the head, shoulders and elbows. This however cannot constitute the primary cause of the difference since the two methods were not statistically different from one another when visual feedback was present. Moreover, the effect of blindfolding for head-pointing was greatest for the horizontal plane, which argues for the existence of an alternative explanation.

The greater undershooting with head pointing in the horizontal plane, compared with the vertical plane, could potentially result from a greater sensitivity to eye movements when making gaze shifts. While it has been clearly documented that small but significant sound localization shifts occur in the opposite direction in response to eccentric gaze (Lewald and Ehrenstein, 1996; Lewald, 1997; Getzmann, 2002), those in the vertical plane are largely dependent on the movement of the head on the neck, whereas horizontal shifts can be augmented with movements from the shoulders, hips and body. One way to address this issue in future work would be to have the subjects perform the sound localization in complete darkness (as opposed to being blindfolded) in order to measure gaze shifts during the localization trials. Alternatively, the increase in undershooting targets when head-pointing in the horizontal plane might also arise due to a shift in the subjective auditory median plane (SAMP) of the head when deprived of visual input. The SAMP might be shifted or biased in the direction of a heard sound while moving toward it, which would lead subjects to undershoot targets due to having the perception of having pointed more eccentrically. This effect has previously been reported (Lewald et al., 2000) where head-pointing to a remembered sound source in darkness produced an undershooting in sound localization responses, that was largely corrected, as in the present study, when laser-pointed feedback of the objective median plane of the head was available. It is thus possible that the visual feedback provided by the laser pointer counteracts the manifestation of such a shift. The lesser impact of blindfolding on hand-pointing on the other hand, could potentially be due to the higher reliability of proprioceptive signals from the arm and hand compared to those provided by the vestibular and head/ neck muscle proprioceptive signals when localizing with the head. Since the present study was not specifically designed to address these issues, further experiments are required in order to fully answer such questions.

A potential caveat of the current experimental design relates to the use of the laser pointer, in that it may have provided a form of super-accurate feedback that is not normally available. This means that the subjects' performance in the non-blindfolded conditions might be better than otherwise expected. While the use of the laser pointing here also served as a means for the experimenter to record the data, future studies may consider alternative recording methods to eliminate this possible bias. Although the average localization error recorded here with the laser pointer when hand-pointing in the horizontal plane (6.36°) does not appear to be markedly better than those previously obtained without the added visual feedback provided by a laser pointer (e.g., Gougoux et al., 2005: 7.61°), future within-experiment control conditions would be best suited to address this issue. Lastly, also unclear at this point is whether it is specifically the localization response that is affected by removal of visual input, or whether the spatial percept itself is also affected. Further experimentation with auditory spatial tasks that do not require an overt motor response (e.g., sound source discrimination tasks) would likely provide valuable insights into this issue.

### Implications for studies with the blind

The present findings demonstrate the effect of performing sound localization tasks while blindfolded and provide compelling evidence that it significantly reduces performance, and does so predominantly under particular circumstances. This observation raises important implications for studies comparing the sound localization abilities of sighted and blind individuals, as the present data argue that specific methodologies should be avoided when doing so. Specifically, in light of the present findings, the use of head-pointing procedures to localize sounds should be avoided, particularly for investigations interested in the horizontal plane. This is particularly important when considering that this discrepancy between pointing methods in the horizontal plane was not found for blind individuals (Haber et al., 1993).

### Conflict of interest statement

The authors declare that the research was conducted in the absence of any commercial or financial relationships that could be construed as a potential conflict of interest.
